# Personalized Medicine in Urolithiasis: AI Chatbot-Assisted Dietary Management of Oxalate for Kidney Stone Prevention

**DOI:** 10.3390/jpm14010107

**Published:** 2024-01-18

**Authors:** Noppawit Aiumtrakul, Charat Thongprayoon, Chinnawat Arayangkool, Kristine B. Vo, Chalothorn Wannaphut, Supawadee Suppadungsuk, Pajaree Krisanapan, Oscar A. Garcia Valencia, Fawad Qureshi, Jing Miao, Wisit Cheungpasitporn

**Affiliations:** 1 Department of Medicine, John A. Burns School of Medicine, University of Hawaii, Honolulu, HI 96813, USA; noppawit@hawaii.edu (N.A.); carayang@hawaii.edu (C.A.); kbvo@hawaii.edu (K.B.V.); cwanna@hawaii.edu (C.W.); 2 Division of Nephrology and Hypertension, Department of Medicine, Mayo Clinic, Rochester, MN 55905, USA; supawadee.sup@mahidol.ac.th (S.S.); garciavalencia.oscar@mayo.edu (O.A.G.V.); qureshi.fawad@mayo.edu (F.Q.); miao.jing@mayo.edu (J.M.); cheungpasitporn.wisit@mayo.edu (W.C.); 3 Chakri Naruebodindra Medical Institute, Faculty of Medicine Ramathibodi Hospital, Mahidol University, Samut Prakan 10540, Thailand; 4 Division of Nephrology, Department of Internal Medicine, Faculty of Medicine, Thammasat University, Pathum Thani 12120, Thailand

**Keywords:** personalized medicine, chatbots, oxalate food content, accuracy, kidney stone, hyperoxaluria, oxalate nephropathy, urolithiasis, nephrolithiasis

## Abstract

Accurate information regarding oxalate levels in foods is essential for managing patients with hyperoxaluria, oxalate nephropathy, or those susceptible to calcium oxalate stones. This study aimed to assess the reliability of chatbots in categorizing foods based on their oxalate content. We assessed the accuracy of ChatGPT-3.5, ChatGPT-4, Bard AI, and Bing Chat to classify dietary oxalate content per serving into low (<5 mg), moderate (5–8 mg), and high (>8 mg) oxalate content categories. A total of 539 food items were processed through each chatbot. The accuracy was compared between chatbots and stratified by dietary oxalate content categories. Bard AI had the highest accuracy of 84%, followed by Bing (60%), GPT-4 (52%), and GPT-3.5 (49%) (*p* < 0.001). There was a significant pairwise difference between chatbots, except between GPT-4 and GPT-3.5 (*p* = 0.30). The accuracy of all the chatbots decreased with a higher degree of dietary oxalate content categories but Bard remained having the highest accuracy, regardless of dietary oxalate content categories. There was considerable variation in the accuracy of AI chatbots for classifying dietary oxalate content. Bard AI consistently showed the highest accuracy, followed by Bing Chat, GPT-4, and GPT-3.5. These results underline the potential of AI in dietary management for at-risk patient groups and the need for enhancements in chatbot algorithms for clinical accuracy.

## 1. Introduction

Nephrolithiasis, often known as kidney stones, is one of the most prevalent urologic conditions, experiencing a significant global increase in both incidence and prevalence, rising from 3.2% to 11% between 1976 and 2018 [1,2,3,4,5]. The clinical manifestations can be varied from asymptomatic cases to those with severe and recurrent symptoms, potentially leading to impaired kidney function. The recurrent progression of kidney stones is significantly influenced by clinical risk factors and lithogenic environments, including metabolic changes with diet and medication [4,6]. Kidney stones are a chronic condition that negatively effects the quality of life of patients in various ways, primarily due to hospitalizations and renal colic’s [7]. Kidney stone patients experience severe recurrent pain during renal colic’s, which can sometimes require surgical interventions. They also face difficulties in following prevention regimens and attending frequent medical appointments. These factors disrupt their normal life activities, work and family time, and sleep patterns. Moreover, patients suffer from psychological distress, such as fear of recurrence or worsening of their condition, and social well-being, including feelings of isolation, as well as problems with intimacy [7,8].

Since calcium oxalate stones, including monohydrate and dihydrate, have been identified as the most common type based on stone composition analysis data [9,10,11], the relationship between dietary habits and the formation of oxalate stones is noteworthy, particularly in the context of calcium oxalate stones, to prevent further stone formation and recurrent disease. 

Oxalate, a compound found in various foods, especially in plants [12], can contribute to stone formation [13]. Dietary oxalate mainly influences the concentration of oxalate in their urine [14]. One of the strategies to prevent calcium oxalate kidney stones is to limit the intake of oxalate-rich foods, along with ensuring adequate calcium intake to bind oxalate in the gastrointestinal tract and reduce its absorption [15]. However, there is no clear evidence-based guideline on how much oxalate intake per day is recommended or optimal for kidney stone prevention. For individuals prone to kidney stones, including those with hyperoxaluria or a history of calcium oxalate stones, healthcare professionals may recommend limiting dietary oxalate intake, which is to aim for a daily oxalate intake below 40 to 50 mg [16,17]. Moreover, when a diet contains more than 50 mg of oxalate per day, the absorption of dietary oxalate increases more steeply, which can further elevate urinary oxalate levels [13]. Thus, managing oxalate intake through dietary adjustments becomes crucial in preventing urolithiasis [18]. Understanding the oxalate content in food is essential for individuals susceptible to calcium oxalate stones, hyperoxaluria, or oxalate nephropathy. Certain foods, such as spinach, rhubarb, and beets, are known to be rich in oxalate and may contribute to stone formation [19]. However, this dietary restriction requires accurate information about the oxalate content in various foods, making it imperative to explore the potential role of artificial intelligence (AI) models in aiding dietary planning. 

With the exponential growth of technology and AI, AI chatbots have the potential to provide information on various parts of medical health. Integrating chatbots might assist in improving the patient care process by identifying risks, enhancing the decision-making process, and providing personalized patient education [20,21]. Several studies have shown that implementing chatbot intervention can encourage physical health, including physical activity and healthy dietary practices [22,23]. Qarajeh et al. [24] demonstrated the potential of chatbots to assist chronic kidney disease (CKD) patients in managing their dietary restrictions by giving information on high potassium and phosphorus food content with an accuracy of more than 70%.

Therefore, AI models may be helpful with oxalate dietary planning in various ways. They can inform patients about oxalate-specific diets and assist healthcare practitioners with dietary evaluation. AI chatbots like ChatGPT [25], Bard AI [26], and Bing Chat [27] are examples of advanced technologies that can offer new solutions for oxalate dietary management. Generative AI models can create new content by learning from large datasets. They can understand context, predict sequences, and generate relevant information. These models have many potential uses, especially in healthcare fields [28,29,30]. ChatGPT-3.5 and ChatGPT-4 are models from OpenAI that can perform various tasks from information retrieval to problem-solving [25,31,32,33,34]. Bard AI is a model that is capable of understanding and generating narratives, creating new plot points, characters, and dialogue that fit a story [26]. Bing Chat is a model from Microsoft that is designed for web-based interactions. It can produce brief and straightforward responses, which is ideal for situations that require quick information retrieval [27]. However, these models need to be tested for their accuracy, reliability, and effectiveness before they are used in healthcare settings [24,35]. 

The potential applications of these AI models in the realm of oxalate dietary planning are diverse and impactful, ranging from patient education to aiding healthcare professionals in dietary evaluation. However, before integrating these models into practical healthcare settings, a thorough evaluation of their effectiveness, precision, and reliability is essential.

The study aims to assess the efficacy of four AI models—ChatGPT 3.5, ChatGPT 4, Bard AI, and Bing Chat—in discerning the oxalate content in foods. This is a crucial consideration for individuals aiming to prevent calcium oxalate urolithiasis [18]. This study is the first to compare the reliability of the four different AI chatbots in categorizing foods based on their oxalate content, which could be potentially integrated into a real-world clinical practice among patients with urolithiasis, who require a specific dietary restriction. We focused on dietary oxalate restriction, in the setting of hyperoxaluria, calcium oxalate renal stones or oxalate nephropathy, as a prototype. The study used a large dataset of various food items to evaluate the AI chatbots’ accuracy, as well as pairwise comparisons between chatbots. We expect to underline the potential of AI chatbots in dietary management and the need for refinements in chatbots algorithms for clinical application.

## 2. Materials and Methods

**Figure 1:**
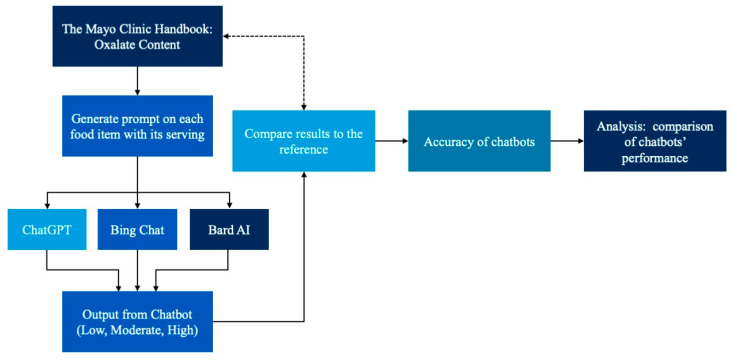
Flowchart of study methods.

We used the Mayo Clinic Oxalate Diet Handbook as the reference to determine the oxalate content of 539 different foods. We classified the foods into three categories based on their dietary oxalate content per serving: low (<5 mg), moderate (5–8 mg), and high (>8 mg) [19,36]. We used the identical prompt to ask four AI chatbots (ChatGPT 3.5, ChatGPT 4, Bard AI, and Bing Chat) to assign each food into high, moderate and low oxalate content, given the provided definition and the serving size. The following prompt was utilized: “Given oxalate content definition as follows: High: >8 mg of oxalate per serving, Moderate: 5–8 mg of oxalate per serving, and Low: <5 mg of oxalate per serving. Please classify the following food by their oxalate content as low, moderate, or high: Considering one serving is equal to _. Is _ considered a low, moderate, or high oxalate food?”. Categorical outputs provided by chatbots as low, moderate, or high oxalate food categories were counted and collected as final answers, regardless of the amount per serving of food items that chatbots can describe. We conducted this study in November 2023. The standardized approach was applied in all four chatbots, including ChatGPT-3.5, from 5–13 November 2023, ChatGPT-4 from 10–14 November 2023, Bard AI from 5–12 November 2023, and Bing Chat from 5–13 November 2023. We collected the chatbot outputs and compared them with the reference values from the Mayo Clinic Diet Handbook to calculate their accuracy rates. We collected the chatbot responses and compared them with the reference groups from the Mayo Clinic Diet Handbook to determine each chatbot’s accuracy in classifying dietary oxalate content. The study methodology is illustrated in Figure 1. Institutional Review Board approval was waived because this study did not involve human subjects or data. The processed dataset is available as a supplementary table (Supplementary Table S1).

### Statistical Analysis

We tested the difference in accuracy across the four AI chatbots using Cochran’s Q test. We tested the difference in accuracy between each pair of AI chatbots using McNemar’s test. We performed a stratified analysis based on dietary oxalate content groups. We considered *p* < 0.05 statistically significant. We performed all statistical analyses using SPSS statistics version 26 (IBM Corp, Armonk, NY, USA).

## 3. Results

Of the 539 foods tested in this study, 277 (51%), 83 (15%), and 179 (33%) were considered low, moderate and high dietary oxalate content. Bard had the highest accuracy in correctly classifying dietary oxalate content (84%, n = 454), followed by Bing Chat (60%, n = 325), ChatGPT-4 (52%, n = 280), and ChatGPT-3.5 (49%, n = 266) (Table 1). 

**Figure 2:**
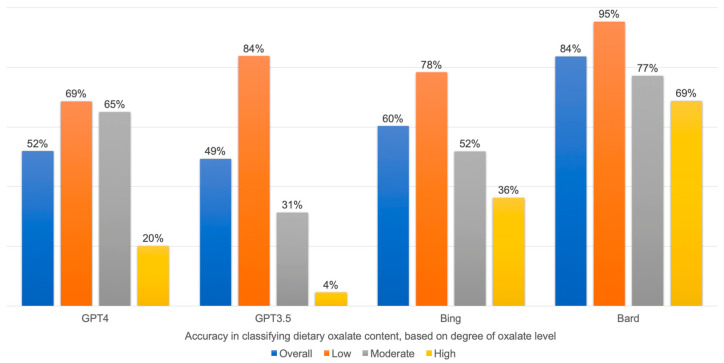
AI chatbot accuracy in classifying dietary oxalate content based on degree of dietary oxalate content.

The accuracy of all chatbots decreased based on the higher degree of dietary oxalate content categories. The accuracy of GPT-4 ranged from 20% to 69%, GPT-3.5 from 4% to 84%, Bing Chat from 36% to 78%, and Bard from 69% to 95% in high to low dietary oxalate content categories, respectively (Table 1 and Figure 2). 

There was a significant difference in the accuracy of classifying overall food items across all four chatbots and when stratified by dietary oxalate content categories (*p* < 0.001). Bard had the highest accuracy in classifying dietary oxalate content in all dietary oxalate categories. In analysis of overall food items, pairwise comparison demonstrated a significant difference in accuracy between all pairs of chatbots, except between GPT-4 and GPT-3.5 (Table 2). However, GPT-4 and GPT-3.5 were significantly different in three subcategories. For the low and high oxalate content groups, all six pairs of chatbots had significant differences, with *p*-values less than 0.001, except for GPT4 and Bing, which had a significant *p*-value of 0.001. For the moderate oxalate group, significant differences were found among ChatGPT-4 and ChatGPT-3.5 (*p* < 0.001), ChatGPT-3.5 and Bing Chat (*p* = 0.01), ChatGPT-3.5 and Bard (*p* < 0.001) and Bing Chat and Bard (*p* = 0.001). However, there was no significant difference between ChatGPT-4 and Bing Chat (*p* = 0.14) or between ChatGPT-4 and Bard (*p* = 0.16) (Table 2).

## 4. Discussion

AI has revolutionized the medical fields, such as medical genetics [37,38], diagnostic investigations [39,40,41], pharmacology [42,43], and clinical nutrition [44,45], especially in providing support for special diet restrictions [24,46]. One of the applications of AI is to help people who need to limit their oxalate intake, which is a common cause of kidney stones. Some AI platforms, such as ChatGPT, Bing Chat, and Bard AI, can offer guidance and suggestions for oxalate restriction diet based on natural language processing and machine learning.

We compared the performance of four promising chatbots: ChatGPT 3.5, ChatGPT 4, Bard AI, and Bing Chat on the novel task of identifying the oxalate content of different foods. This is the first study to evaluate chatbot performance on oxalate content identification, which is a useful task for people who need to monitor their oxalate intake. We found that chatbots relatively performed better on low oxalate content foods than on moderate or high oxalate foods. 

The results of this study suggest that Bard AI is the most reliable chatbot for detecting oxalate content in food items, followed by Bing Chat. ChatGPT-4 and ChatGPT-3.5 performed poorly and showed no overall significant difference between them. Regarding the subgroup analysis, the results consistently showed that Bard AI had the highest accuracy in all three categories, followed by ChatGPT-4 as the second-best chatbot in the moderate category, ChatGPT-3.5 as the second-best chatbot in the low category and Bing Chat as the second-best AI chatbot in the high category. The differences between the chatbots were statistically significant in most cases, except for a few pairs in the moderate group, between ChatGPT-4 versus Bing Chat, and ChatGPT-4 versus Bard, that did not show significant differences. A relatively lower sample size in the moderate group at 83 (15%) from 539 items might not yield enough power to demonstrate statistical differences in these subgroups. However, none of the chatbots achieved satisfying accuracy, especially the accuracy of ChatGPT-3.5, ChatGPT-4, and Bing, which is notably below 40% in identifying high oxalate foods. This is critical when applying chatbots on food-related information, particularly in high-risk patients who are prone to developing oxalate stones. The use of chatbots in this context requires caution to standard guidelines or supervision from healthcare providers.

Compared to the related studies, a study by Qarajeh et al. [24] compared the performance of the same four chatbots in classifying the potassium and phosphorus content of 240 different food items into either low or high categories. They similarly found that Bard AI had the highest accuracy, ranging from 79% to 100%, followed by Bing Chat, ChatGPT-4 and ChatGPT-3.5. Their results indicated that all four chatbots achieved higher overall accuracy than this study. However, they had a fewer sample size at 240 food items and less strict answer criteria, which was either low or high. We had 539 food items and three answer categories, including low, moderate, and high, which were likely related to the inconsistency of outcomes. We noticed that the chatbots often repeated their previous answers or misclassified foods into the wrong oxalate groups. Moreover, ChatGPT-3.5 and ChatGPT-4 had long processing times when given more than 300 prompts. Therefore, increasing the number of the sample size was supposed to improve the statistical power, but it may affect the chatbot accuracy negatively. In contrast to the current findings, our previous study compared the accuracy of AI chatbots, including ChatGPT-3.5, Bing Chat and Bard AI, in performing a literature search in the field of Nephrology. We observed that ChatGPT provided the highest proportion of correct references (38%), followed by Bing Chat (28%) and Bard (3%). This result was completely different from our current study, where Bard had the highest accuracy, followed by Bing Chat and ChatGPT, respectively [47]. For the emergency medicine setting, Zúñiga Salazar et al. conducted a study to evaluate the accuracy of three AI chatbots, including ChatGPT-3.5, Google Bard AI, and Bing Chat, in differentiating between medical emergency and non-emergency scenarios, based on the questions asked by patients from the online forum on Reddit. The results showed that Google Bard had the highest accuracy in detecting the true medical emergency (87%), followed by ChatGPT-3.5 (77%) and Bing Chat (82%), but the differences were not statistically significant. On the other hand, ChatGPT-3.5 had a slightly higher accuracy (36%) in detecting the non-emergency situation than Bard AI (33%) and Bing Chat (26%). The study indicated that the AI chatbots tended to overclassify the scenarios as emergencies and under-classify them as non-emergencies, compared to human reviewers [48]. 

None of these chatbots achieved excellent performance, and AI chatbots still need further improvement before they can be considered as reliable sources in the healthcare field. The different outcomes among the chatbots reflect the strengths, weaknesses, and limitations of chatbots in different scenarios. It could be attributed to the quality and quantity of the database available in each chatbot, their underlying architectures, training data, and natural language understanding capabilities. The complexity, variability, and difficulty measuring of foods also plays a role in how the chatbots interpret and categorize each food item.

This study has implications for the development and evaluation of chatbots for health-related purposes, especially for people who need to monitor their oxalate intake. We believe that we are the first study that compares the accuracy of the four chatbots to provide oxalate content information. This study had strengths, such as using the four well-known AI chatbots as the subjects, having a large number of the 539 food items to test, and using a reliable reference is the Mayo Clinic Oxalate Diet Handbook. We used a uniform method to evaluate each chatbot according to predefined criteria. The study also performed robust statistical analysis to examine the differences in accuracy across chatbots and between each pair of chatbots, as well as a stratified analysis based on oxalate content groups. We ensured the consistency of the results by conducting the assessment during a specific period for each chatbot, avoiding potential variations due to chatbot updates or changes.

Several limitations and errors that could compromise their usefulness and credibility were identified. One notable limitation observed in our study pertains to the tendency of the AI chatbot, specifically ChatGPT, to provide similar responses as previous outputs. This recurrent pattern of identical responses across diverse food queries raises concerns about the model’s ability to generate nuanced and context-specific information. While the AI chatbot demonstrates commendable proficiency in certain aspects, the observed repetition underscores the need for further refinement and enhancement to ensure the accuracy and diversity of responses, especially in scenarios involving repetitive questioning on distinct food items. We did not simulate the real-life situation that patients would encounter when they have various types of food on each meal. Instead, we assigned food items based on the Mayo Clinic Nutrition Handbook, which group foods into categories (Supplementary Table S1). This may have resulted in a bias, as food items with similar names in the same category were more likely to be prompted consecutively. Since AI chatbots tend to repeat the answer based on the previous food item, this may have either negatively or positively affected the accuracy of our chatbot. Therefore, using AI chatbots in a real-world setting might yield different accuracy levels and outcomes. We recommend that future studies using AI chatbot should run the prompts separately in a new chat for each item. Secondly, all AI chatbots sometimes misclassified food items into the wrong oxalate groups, even when they correctly identified the oxalate amount per serving. We did not request the exact amount of oxalate content in our prompt; thus, we accepted the chatbots’ responses in categorical outcomes as low, moderate, or high oxalate food categories. These were defined as the final answers we used for the analysis, even though the chatbots sometimes provided the oxalate amount per serving of food items. Using the oxalate content in milligrams per serving as a criterion to evaluate the chatbots output could lead to different levels of accuracy. Thirdly, we found that ChatGPT, both GPT-3.5 and GPT-4, experienced significant delays in processing when given more than 300 prompts, and that starting a new chat was more efficient than continuing the existing one. Moreover, the potential changes in the chatbots’ algorithm over time could affect their performance and consistency. Since there is no clear recommendation for dietary oxalate intake and no universal classification of oxalate food content, a clinical practice guideline that defines these important numbers is needed. Lastly, the study did not examine the clinical outcomes of oxalate restriction with the assistance of AI chatbots. This is an important area for future investigation, after the AI chatbots are validated to have satisfactory accuracy. 

Acknowledging and addressing these limitations is crucial for fostering confidence in the reliability and effectiveness of AI chatbots as tools for dietary guidance, particularly in the context of managing oxalate intake for individuals with renal conditions. Future iterations and advancements in AI technology should aim to mitigate these limitations and enhance the adaptability and specificity of responses, ultimately contributing to the utility of such tools in personalized dietary recommendations for individuals with specific health considerations. 

These findings imply that AI chatbots have the potential to transform the medical field, but they also pose significant ethical challenges [49,50,51,52]. Healthcare professionals and policymakers need to carefully weigh the benefits and risks of AI integration and consider the ethical implications of their decisions. AI chatbots still have room for improvement in generating accurate and reliable references for medical education and research [53]. They also highlight the need for users to critically evaluate the sources and quality of the information provided by chatbots, and to verify them with other authoritative sources. Furthermore, they indicate that future studies should investigate other aspects of chatbot performance, such as their ability to handle different medical topics, their responsiveness and efficiency, or their impact on clinical decision-making and patient outcomes.

## 5. Conclusions

This study indicates that none of the chatbots had flawless accuracy, but Bard AI is the most reliable chatbot for detecting oxalate content in food items, followed by Bing Chat and ChatGPT. ChatGPT-4 and ChatGPT-3.5 showed no overall significant difference between them. The differences among the chatbots could be explained by factors such as database quality and quantity, architecture, and natural language understanding capabilities. The effects of AI-assisted diet intervention on clinical outcomes require further investigation.

## Figures and Tables

**Table 1:** The overall accuracy of AI chatbots in classifying dietary oxalate content.

Oxalate Content	GPT-4	GPT-3.5	Bing	Bard	*p*-Value
Overall (n = 549)	280 (52%)	266 (49%)	325 (60%)	451 (84%)	<0.001
Low (n = 277)	190 (69%)	232 (84%)	217 (78%)	264 (95%)	<0.001
Moderate (n = 83)	54 (65%)	26 (31%)	43 (52%)	64 (77%)	<0.001
High (n = 179)	36 (20%)	8 (4%)	65 (36%)	123 (69%)	<0.001

**Table 2:** *p*-value from pairwise comparison of accuracy between AI chatbots in classifying dietary oxalate contents.

Pairwise Comparison	Overall	Dietary Oxalate Content		
		Low	Moderate	High
GPT-4 vs. GPT-3.5	0.298	<0.001	<0.001	<0.001
GPT-4 vs. Bing	0.003	0.001	0.14	<0.001
GPT-4 vs. Bard	<0.001	<0.001	0.16	<0.001
GPT-3.5 vs. Bing	<0.001	<0.001	0.01	<0.001
GPT-3.5 vs. Bard	<0.001	<0.001	<0.001	<0.001
Bing vs. Bard	<0.001	<0.001	0.001	<0.001

## Data Availability

Data Availability Statements are available in the original publication, reports, and preprints that were cited in the reference.

## Supplementary Materials

The following supporting information can be downloaded at: https://www.mdpi.com/article/10.3390/jpm14010107/s1, Table S1: Dataset of AI chatbots’ output on oxalate content in various food items.
