# Supplementary material for: Personalized Medicine in Urolithiasis: AI Chatbot-Assisted Dietary Management of Oxalate for Kidney Stone Prevention
Source: J Pers Med. 2024 Jan 18;14(1):107. doi: 10.3390/jpm14010107 (PMC10817681; doi:10.3390/jpm14010107)
Supplement: Supplementary file 1 [file jpm-14-00107-s001.zip › jpm-2815512-supplementary.pdf]

Table S1

FOOD ITEMS	SERVING SIZE	OXALATE CATEGORY	OXALATE VALUE	reference	GPT4 (11/10/23- 11/14/23)	GPT3.5 (11/5/23 - 11/13/23)	Bing (11/5/23 - 11/12/23)	Bard (11/5/23 - 11/13/23)
Avocados	1 fruit	Very high	19 mg	3	1	1	3	3
Dates	1 date	Very high	24 mg	3	2	1	3	3
Grapefruit	1/2 fruit	Very high	12 mg	3	2	1	1	3
Kiwi	1 fruit	Very high	16 mg	3	3	1	2	2
Orange	1 fruit	Very high	29 mg	3	1	1	3	3
Raspberries	1 cup	Very high	48 mg	3	2	1	3	3
Tangerine	1 fruit	High	10 mg	3	1	1	1	3
Figs	1 medium fig	Moderate	9 mg	2	2	3	3	2
Apple Sauce	1 cup	Low	2 mg	1	1	1	~~1~~	1
Banana	1 fruit	Low	3 mg	1	1	1	1	1
Blackberries	1/2 cup	Low	2 mg	1	2	0	1	1
Blueberries	1/2 cup	Low	2 mg	1	2	1	1	1
Cherries	1 cup	Low	3 mg	1	1	1	1	1
Limes	1/2 fruit	Low	3 mg	1	3	1	0	1
Pears	1 fruit	Low	2 mg	1	2	1	1	1
Pineapple	1 cup	Low	4 mg	1	2	1	1	1
Raisins	1 oz or 1 small sna	Low	3 mg	1	3	3	1	1
Strawberries	1/2 cup	Low	2 mg	1	2	1	1	1
Apples	1 fruit	Low	1 mg	1	1	1	1	1
Apricots	1 fruit	Little or none	0 mg	1	1	1	1	1
Cantaloupe	1/4 melon	Little or none	1 mg	1	1	1	1	1
Grapes	1/2 cup or 16 seec	Little or none	1 mg	1	2	1	1	1
Honeydew Melon	1 cup	Little or none	1 mg	1	1	1	1	1
Lemons	1 wedge	Little or none	1 mg	1	3	1	0	1
Mango	1 fruit	Little or none	1 mg	1	2	1	1	1
Nectarine	1 medium fruit	Little or none	1 mg	1	1	1	1	1
Papaya	1 fruit	Little or none	1 mg	1	1	1	1	1
Peaches	1 fruit	Little or none	1 mg	1	2	1	1	1
Plantain	1 medium	Little or none	1 mg	1	2	1	1	1
Plums	1 fruit	Little or none	1 mg	1	2	1	1	1
Watermelon	1 slice	Little or none	1 mg	1	1	1	1	1
Canned Pineapple	1/2 cup	Very high	24 mg	3	2	0	2	3
Canned Cherries	1/2 cup	Moderate	7 mg	2	1	0	2	2
Cranberry Sauce	1/2 cup	Low	2 mg	1	2	0	2	1
Canned Pears	1/2 cup	Little or none	1 mg	1	2	1	1	1
Canned Peaches	1/2 cup	Little or none	1 mg	1	2	1	1	1
Fruit Cocktail	1/2 cup	Little or none	1 mg	1	1	1	1	1
Dried Figs	5 pieces/fruits	Very high	24 mg	3	3	0	2	3
Dried Pineapples	1/2 cup	Very high	30 mg	3	3	0	2	3
Dried Prunes	1/4 cup or 5 prune	High	11 mg	3	2	2	2	3
Dried Apples	1 cup or 13 rings	Low	2 mg	1	1	0	1	1
Dried Apricots	1 cup of halves	Low	3 mg	1	2	3	1	1
Dried Cranberries	1/2 cup	Little or none	1 mg	1	2	0	1	1
Bamboo Shoots	1 cup	Very high	35 mg	3	1	1	2	3
Beets	1/2 cup	Very high	76 mg	3	3	1	2	3
Fava Beans	1/2 cup	Very high	20 mg	3	2	2	2	3
Navy Beans	1/2 cup	Very high	76 mg	3	3	1	2	3
Okra	1/2 cup	Very high	57 mg	3	3	1	2	3
Olives	~10 olives	Very high	18 mg	3	1	1	1	1
Parsnip	1/2 cup	Very high	15 mg	3	2	1	2	1
Red Kidney Beans	1/2 cup	Very high	15 mg	3	3	2	1	3
Refried Beans	1/2 cup	Very high	16 mg	3	3	1	2	1
Rhubarb	1/2 cup	Very high	541 mg	3	3	3	2	3
Rutabaga	1/2 cup	Very high	31 mg	3	2	1	2	3
Spinach, cooked	1/2 cup	Very high	755 mg	3	3	3	3	3
Spinach, raw	1 cup	Very high	656 mg	3	3	3	3	3
Tomato Sauce	1/2 cup	Very high	17 mg	3	2	1	2	1
Turnip	1/2 cup, mashed	Very high	30 mg	3	1	1	2	3
Yams	1/2 cup, cubed	Very high	40 mg	3	2	1	2	3
Carrots, raw	1/2 large carrot	Very high	15 mg	3	1	1	1	1
Celery, cooked	1 cup	High	10 mg	3	1	1	1	1
Collards	1 cup	High	10 mg	3	3	1	1	1
Artichokes	1 small bud	Moderate	5 mg	2	2	1	1	1
Asparagus	4 spears	Moderate	6 mg	2	2	1	1	2
Carrots, cooked	1/2 cup, sliced	Moderate	7 mg	2	1	1	1	2
Hot Chili Peppers	1/2 cup	Moderate	5 mg	2	2	1	1	2
Mixed Vegetables, frozen	1 cup	Moderate	5 mg	2	2	1	1	2
Oriental Vegetables, frozen	1/2 cup	Moderate	6 mg	2	2	1	2	2

Soybeans	1 cup	Very high	96 mg	2	3	2	1	2
String Beans	1/2 cup	Moderate	9 mg	2	1	1	1	2
Tomato	1 medium	Moderate	7 mg	2	2	1	1	2
Brussel Sprouts	1/2 cup, frozen	Very high	17 mg	3	2	1	2	1
Celery, raw	1/2 cup	Very high	19 mg	3	1	1	1	1
Kale	1 cup	Low	2 mg	1	2	1	1	1
Mung Beans	1/2 cup	Moderate	8 mg	2	3	1	2	1
Mustard Greens	1 cup	Low	4 mg	1	3	2	1	1
Sea Vegetables	1 cup	Low	3 mg	1	1	1	1	1
Alfalfa Sprouts	1/2 cup	Little or none	0 mg	1	1	1	1	1
Bok Choy (Chinese Cabbag	1 cup, raw	Little or none	1 mg	1	1	1	1	1
Broccoli	1/2 cup, chopped	Moderate	6 mg	2	1	1	1	1
Cabbage	1/2 cup	Little or none	1 mg	1	1	1	1	1
Cauliflower	1/2 cup, cooked	Little or none	1 mg	1	1	1	1	1
Chives	1 tsp	Little or none	0 mg	1	1	1	1	1
Corn	1/2 cup	Little or none	1 mg	1	2	1	1	1
Cucumber	1/4 cucumber	Little or none	1 mg	1	1	1	1	1
Endive	1/2 cup	Little or none	0 mg	1	1	1	1	1
Green Pepper	1/2 cup	Little or none	5 mg	1	1	1	1	1
Iceberg Lettuce	1 cup	Little or none	0 mg	1	1	1	1	1
Mushrooms	1 mushroom	Little or none	0 mg	1	2	1	1	1
Onions	1 small onion	Little or none	1 mg	1	1	1	1	1
Peas	1/2 cup	Little or none	0 mg	1	2	1	1	1
Pickles	10 count	Little or none	0 mg	1	1	1	1	1
Radish	1 cup	Little or none	1 mg	1	2	1	1	1
Romaine Lettuce	1/2 cup	Little or none	1 mg	1	1	1	1	1
Scallions	1/2 cup	Little or none	0 mg	1	1	1	1	1
Sauerkraut	4 oz or 1/2 cup	Little or none	4 mg	1	2	2	1	2
Water Chestnuts	4 water chestnuts	Little or none	1 mg	1	1	1	1	1
Yellow Squash	1/2 cup	Low	4 mg	1	1	1	1	1
Zucchini	1/2 cup	Little or none	1 mg	1	1	1	1	1
French Fries, homemade o	1 oz	Very high	51 mg	3	2	1	3	3
Baked Potato with Skin	1/3 cup	Very high	97 mg	3	2	1	1	3
Mashed Potatoes	1 cup	Very high	29 mg	3	2	2	2	3
Potato Chips	1 cup	Very high	21 mg	3	2	3	3	3
Potato Salad	1 cup	Very high	17 mg	3	2	2	2	3
Sweet Potatoes	1 cup	Very high	28 mg	3	2	1	1	3
Homemade Cream Sauce	1 cup	Low	3 mg	1	1	3	1	1
Coffee Creamer	1 tbsp	Little or none	0 mg	1	1	1	1	1
Non-Dairy Creamer	1 tbsp	Little or none	0 mg	1	1	1	1	1
Sour Cream	1 tbsp	Little or none	0 mg	1	1	1	1	1
Vanilla Ice Cream	1/2 cup	Little or none	0 mg	1	1	2	1	1
Ice Cream, light	1/2 cup	Little or none	0 mg	1	1	2	1	1
Ice Cream, non-fat	1/2 cup	Little or none	0 mg	1	1	2	1	1
Plain Yogurt	1 cup	Low	2 mg	1	1	1	1	1
Yogurt with Fruit	8 oz	Little or none	1 mg	1	2	1	1	1
Yogurt with Fruit, non-fat	8 oz	Little or none	1 mg	1	2	1	1	1
Frozen Yogurt	1/2 cup	Little or none	1 mg	1	2	1	1	1
Frozen Yogurt, low fat	1/2 cup	Little or none	1 mg	1	2	1	1	1
American Cheese	1 slice	Little or none	0 mg	1	1	1	1	1
Cheddar Cheese	1 slice	Little or none	0 mg	1	1	1	1	1
Cheese, low fat	1 slice	Little or none	0 mg	1	1	1	1	1
Cottage Cheese	1/2 cup	Little or none	0 mg	1	1	1	1	1
Cottage Cheese, low fat	1 cup	Little or none	1 mg	1	1	1	1	1
Cottage Cheese, fat free	1/2 cup	Little or none	0 mg	1	1	1	1	1
Mozzarella Cheese	1 oz	Little or none	0 mg	1	1	1	0	1
Eggs	1 medium	Little or none	0 mg	1	1	1	0	1
Egg Beaters	4 oz	Little or none	0 mg	1	1	1	0	1
Cream Cheese	1 oz	Little or none	0 mg	1	1	1	1	1
Cream Cheese, fat free	1 oz	Little or none	1 mg	1	1	1	1	1
Cream Cheese, low fat	1 oz	Little or none	1 mg	1	1	1	1	1
Butter, salted	1 pat	Little or none	0 mg	1	1	1	0	1
Chocolate Milk	1 cup	Moderate	7 mg	2	2	1	0	1
Powdered Milk	1 envelope	Low	3 mg	1	1	1	0	1
Fat Free Milk	1 cup	Little or none	1 mg	1	1	1	1	1
1% Milk	1 cup	Little or none	1 mg	1	1	1	1	1
2% Milk	1 cup	Little or none	1 mg	1	1	1	1	1
Whole Milk	1 cup	Little or none	1 mg	1	1	1	1	1
Buttermilk	1 cup	Little or none	1 mg	1	1	1	1	1

French Toast	2 slices	Very high	13 mg	3	2	1	3	3
Bagel New York	1 bagel	Very high	40 mg	3	2	1	3	3
Whole Wheat English Muffi	1 muffin	High	12 mg	3	2	1	2	2
Pancakes, homemade	4 cakes	High	11 mg	3	2	1	3	3
Pancakes, mix	4 cakes	High	10 mg	3	2	1	3	3
Blueberry Muffins	1 muffin	High	9 mg	3	2	1	1	2
Biscuits, plain or buttermilk	1 biscuit	Moderate	6 mg	2	2	1	2	2
Bran Muffins	1 muffin	Moderate	5 mg	2	2	1	2	2
Bran Muffin, low fat	1 muffin	Moderate	5 mg	2	2	1	2	2
Cracked Wheat Bread	1 slice	Moderate	5 mg	2	2	1	2	2
English Muffin	1 muffin	Moderate	8 mg	2	2	1	2	2
Multi-Grain English Muffin	1 muffin	Moderate	8 mg	2	1	1	2	2
Wheat English Muffin	1 muffin	Moderate	7 mg	2	2	1	2	2
Low Fat Muffins	1 muffin	Moderate	5 mg	2	2	1	0	2
Rye Bread	1 slice	Moderate	7 mg	2	2	1	2	2
Corn Tortillas	1 tortilla	Moderate	7 mg	2	2	1	2	1
Flour Tortillas	1 tortilla	Moderate	8 mg	2	2	1	1	2
White Bread	1 slice	Moderate	5 mg	2	3	1	2	2
Wheat Bran Bread	1 slice	Moderate	7 mg	2	2	1	3	3
Whole Oat Bread	1 slice	Moderate	5 mg	2	2	1	0	1
Whole Wheat Bread	1 slice	Moderate	6 mg	2	2	1	2	2
Plain Bagel	1 bagel	Low	9 mg	1	2	1	2	2
Corn Bread	1 piece	Low	4 mg	1	2	1	2	1
Oatmeal Bread	1 piece	Low	4 mg	1	2	1	1	1
Oat Bran Muffin	1 small muffin	Low	4 mg	1	2	1	1	1
Oat Bran Bread	1 slice	Low	4 mg	1	2	1	2	1
All-Purpose Flour	1 cup	Very high	17 mg	3	2	1	2	3
Brown Rice, cooked	1 cup	Very high	24 mg	3	2	1	2	2
Brown Rice Flour	1 cup	Very high	65 mg	3	2	1	3	3
Buckwheat Groats	1 cup, cooked	Very high	133 mg	3	2	1	2	3
Bulgur, cooked	1 cup	Very high	86 mg	3	2	1	0	3
Corn Grits	1 cup	Very high	97 mg	3	2	1	2	3
Cornmeal	1 cup	Very high	64 mg	3	1	1	1	3
Couscous	1 cup	Very high	15 mg	3	2	1	2	3
Lasagna	1 serving	Very high	23 mg	3	2	2	2	3
Millet, cooked	1 cup	Very high	62 mg	3	2	1	2	2
Miso	1 cup	Very high	40 mg	3	2	1	3	3
Rice Bran	1 cup	Very high	281 mg	3	3	1	3	3
Soy Flour	1 cup	Very high	94 mg	3	3	2	2	3
Wheat Berries	1 cup, cooked	Very high	98 mg	3	3	2	2	3
Whole Grain Wheat Flour	1 cup	Very high	29 mg	3	2	1	1	3
Spaghetti	1 cup, cooked	High	11 mg	3	2	2	1	1
White Rice Flour	1 cup	High	11 mg	3	1	2	3	1
Corn Flour	1 cup	Low	3 mg	1	2	1	1	1
Hummus	1 tbsp	Low	4 mg	1	2	1	2	1
Macaroni & Cheese	1 cup	Low	4 mg	1	2	2	2	1
White Rice, cooked	1 cup	Low	4 mg	1	1	1	1	1
Barley Malt Flour	1 cup	Little or none	0 mg	1	2	1	3	1
Corn Bran	1 cup	Little or none	0 mg	1	2	3	0	1
Flaxseed	1 tbs	Little or none	0 mg	1	3	1	0	1
Oat Bran, raw	1/3 cup	Little or none	0 mg	1	3	2	1	1
Tofu	3.5 oz	Very high	13 mg	3	2	1	3	2
Veggie Burger	1 patty	Very high	24 mg	3	2	1	3	0
Soy Burger	3.5 oz	High	12 mg	3	2	1	3	2
Chicken Nuggets	6 nuggets	Low	3 mg	1	2	2	1	1
Meatballs	2 meatballs	Low	2 mg	1	2	0	1	1
Turkey Dogs	1 dog	Low	3 mg	1	1	0	1	1
Antelope	3 oz	Little or none	0 mg	1	1	1	1	1
Bacon	2 slices	Little or none	0 mg	1	1	1	1	1
Bologna	1 slice	Little or none	0 mg	1	1	1	1	1
Buffalo	3 oz	Little or none	0 mg	1	1	1	1	1
Chicken Dog	1 dog	Little or none	1 mg	1	1	1	1	1
Chicken Liver	3 oz	Little or none	0 mg	1	2	1	1	1
Chicken	3 oz	Little or none	0 mg	1	1	1	1	1
Hot Dogs	1 dog	Little or none	1 mg	1	1	1	1	1
Ham	3 oz	Little or none	0 mg	1	1	1	1	1
Ground Beef	3 oz	Little or none	0 mg	1	1	1	1	1
Lean Hamburger (85%)	3 oz	Little or none	0 mg	1	1	1	1	1
Lean Hamburger (75%)	3 oz	Little or none	0 mg	1	1	1	1	1

Lean Hamburger (90%)	3 oz	Little or none	1 mg	1	1	1	1	1
Liver	3.5 oz	Little or none	0 mg	1	3	1	1	1
Moose	3 oz	Little or none	0 mg	1	1	1	1	1
Pork	5 oz	Little or none	0 mg	1	1	1	1	1
Turkey	5 oz	Little or none	0 mg	1	1	1	1	1
Venison	3 oz	Little or none	0 mg	1	1	1	1	1
Wild Game Meat	3 oz	Little or none	1 mg	1	1	1	1	1
Tuna Salad	1 cup	Moderate	6 mg	2	1	1	1	2
Frozen Fish Sticks	2 sticks	Low	3 mg	1	2	1	1	1
Alaskan King Crab	3 oz or 1/2 leg	Little or none	0 mg	1	1	1	1	1
Bluefish	1 fillet	Little or none	1 mg	1	1	1	1	1
Clams, raw	3 oz	Little or none	0 mg	1	1	1	1	1
Cod, Pacific	3 oz or 1 fillet	Little or none	0 mg	1	1	1	1	1
Cod Liver Fish Oil	1 tsp	Little or none	0 mg	1	1	1	1	1
Flounder	3 oz	Little or none	0 mg	1	1	1	1	1
Haddock	3 oz	Little or none	0 mg	1	1	1	1	1
Halibut	3 oz	Little or none	0 mg	1	1	1	1	1
Herring, Atlantic & Pacific	3 oz	Little or none	1 mg	1	1	1	1	1
Mackerel	3 oz	Little or none	0 mg	1	1	1	1	1
Oysters	3 oz	Little or none	0 mg	1	1	1	1	1
Pollock	3 oz	Little or none	0 mg	1	1	1	1	1
Salmon, all types	4 oz	Little or none	0 mg	1	1	1	1	1
Sardines	1 can or 3.75 oz	Little or none	0 mg	1	1	1	1	1
Shrimp	3 oz	Little or none	0 mg	1	1	1	1	1
Swordfish	1 piece	Little or none	0 mg	1	1	1	1	1
Tuna Fish, in oil	3.5 oz	Little or none	0 mg	1	1	1	1	1
Tuna Fish, in water	3.5 oz	Little or none	0 mg	1	1	1	1	1
Whiting	3 oz	Little or none	0 mg	1	1	1	1	1
Almonds	1 oz or 22 kernels	Very high	122 mg	3	3	1	3	3
Candies with Nuts (e.g., Sn	2 oz	Very high	38 mg	3	3	1	3	3
Cashews	1 oz or 18 kernels	Very high	49 mg	3	3	2	3	2
Peanuts	1 oz	Very high	27 mg	3	3	1	3	2
Pistachios	1 oz or 48 kernels	Very high	14 mg	3	2	1	2	2
Mixed Nuts, with peanuts	1 oz	Very high	39 mg	3	3	2	2	2
Pumpkin Seeds	1 cup, cooked	Very high	17 mg	3	3	2	2	2
Trail Mix	1 oz	Very high	15 mg	3	0	2	2	2
Walnuts	1 cup or 7 nuts	Very high	31 mg	3	2	1	2	3
Pecans	1 oz or 15 halves	High	10 mg	3	2	1	2	1
Sunflower Seeds	1 cup	High	12 mg	3	3	1	1	2
Flaxseed	1 tbsp	Little or none	0 mg	1	3	1	1	1
Brownies	1 oz or 1/2 browni	Very high	31 mg	3	2	2	3	2
Cake, store brand	1 piece	Very high	15 mg	3	2	2	3	2
Cake, homemade	1 piece	Very high	16 mg	3	2	1	0	3
Candies with Nuts (e.g., Sn	2 oz	Very high	38 mg	3	3	2	0	3
Chocolate Syrup	2 tbsp	Very high	38 mg	3	2	1	3	3
Fudge Sauce	2 tbsp	Very high	28 mg	3	2	1	3	3
Cake, low fat only	1 piece	High	11 mg	3	2	2	3	1
Chocolate Chip Cookies, st	1 cookie	High	10 mg	3	2	1	3	2
Chocolate Chip Cookies, lo	1 cookie	Moderate	7 mg	2	2	2	3	2
Milk Chocolate Candies	1 oz	Moderate	5 mg	2	2	1	2	1
Pies, homemade	1 piece or 1/8th of	Moderate	5 mg	2	2	2	3	2
Apple Pie	1 piece or 1/8th of	Moderate	5 mg	2	2	2	2	1
Pudding Popsicle	1 pop	Moderate	5 mg	2	2	1	2	1
Fig Bars	1 cookie	Low	4 mg	1	2	1	3	1
Chocolate Pudding, instant	1/4 box or 1 oz	Low	4 mg	1	2	1	3	1
Oatmeal Cookies, store	1 cookie	Low	4 mg	1	2	2	2	1
Oatmeal Cookie, homemad	1 cookie	Low	2 mg	1	2	2	2	1
Rice Cake	1 cake	Low	4 mg	1	1	1	1	1
Rice Pudding	1/2 cup	Low	2 mg	1	1	2	2	1
Snack Cakes, crème filled	1 cake	Low	3 mg	1	2	3	3	1
Custard	1 cup	Little or none	1 mg	1	1	1	1	1
Jell-O	1 cup	Little or none	1 mg	1	1	1	1	1
Popsicle	1 stick	Little or none	0 mg	1	1	1	1	1
Rice Krispy Treat	1 bar	Little or none	1 mg	1	1	1	1	1
Sherbert	1/2 cup	Little or none	0 mg	1	2	1	1	1
Tapioca Pudding	1/2 cup	Little or none	0 mg	1	1	1	1	1
Vanilla Pudding	1 cup	Little or none	1 mg	1	1	1	1	1
Potato Chips	1 oz	Very high	21 mg	3	2	3	3	2
Tortilla Corn Chips	1 oz	Moderate	7 mg	2	1	3	2	2

Popcorn, oil-popped	1 cup	Moderate	5 mg	2	1	2	1	1
Pretzels, hard & salted	1 oz	Moderate	5 mg	2	2	3	1	2
Fruit Roll-Ups	1 roll	Low	2 mg	1	1	2	1	1
Graham Crackers	1 large rectangle	Low	2 mg	1	2	1	1	1
Popcorn, air-popped	1 cup	Low	4 mg	1	1	1	1	1
Ritz Crackers	5 crackers	Low	3 mg	1	2	1	1	1
Saltines	1 cracker	Little or none	1 mg	1	2	1	1	1
Triscuits	1 cracker	Little or none	1 mg	1	2	1	1	1
Wheat Crackers	1 cracker	Little or none	1 mg	1	2	1	1	1
Wheat Thins, reduced fat	1 cracker	Little or none	1 mg	1	2	1	1	1
Carrot Juice	1 cup	Very high	27 mg	3	2	1	1	3
Hot Chocolate, homemade	1 cup	Very high	65 mg	3	2	1	2	3
Lemonade, frozen from cor	8 oz	Very high	15 mg	3	1	1	2	3
Rice Dream	1 cup	Very high	13 mg	3	1	1	2	3
Tea, brewed	1 cup	Very high	14 mg	3	3	1	2	1
Tomato Juice	1 cup	Very high	14 mg	3	2	1	2	1
V8 Juice	1 cup	Very high	18 mg	3	2	1	2	2
Soy Milk	1 cup	Very high	20 mg	3	2	1	2	1
Prune Juice	1 cup	Moderate	7 mg	2	2	1	1	2
Apple Juice	6 oz	Low	2 mg	1	1	1	1	1
Apricot Juice	1 cup	Low	2 mg	1	1	1	1	1
Coffee Decaf	1 cup	Low	2 mg	1	2	1	1	1
Orange Juice	1 cup	Low	2 mg	1	1	1	1	1
Pineapple Juice	8 oz	Low	3 mg	1	1	1	1	1
Postum, coffee substitute	1 serving	Low	2 mg	1	1	1	1	1
Coffee	1 cup	Low	1 mg	1	2	1	1	1
Gatorade	1 cup	Little or none	0 mg	1	1	1	1	1
Grape Juice	8 oz	Little or none	1 mg	1	2	2	1	1
Grapefruit Juice	8 oz	Little or none	0 mg	1	2	2	2	1
Kool-Aid	1 cup	Little or none	1 mg	1	1	1	1	1
Lemonade, diet	8 oz	Little or none	1 mg	1	1	1	0	1
Mango Juice	8 oz	Little or none	1 mg	1	2	1	1	1
Sodas, all types	8 oz	Little or none	0 mg	1	1	1	1	1
Sweetened Instant Iced Tea	8 oz	Little or none	0 mg	1	3	1	1	1
Water	8 oz	Little or none	0 mg	1	1	1	1	1
Chocolate Milk	1 cup	Moderate	7 mg	2	2	1	1	2
Powdered Milk	1 envelope	Low	3 mg	1	1	1	1	1
Fat Free Milk	1 cup	Little or none	1 mg	1	1	1	1	1
1% Milk	1 cup	Little or none	1 mg	1	1	1	1	1
2% Milk	1 cup	Little or none	1 mg	1	1	1	1	1
Whole Milk	1 cup	Little or none	1 mg	1	1	1	1	1
Beer, regular	1 can	Low	4 mg	1	2	1	1	1
Beer, light	1 can	Low	3 mg	1	2	1	1	1
Red Wine	4 oz	Little or none	1 mg	1	2	1	1	1
White Wine	4 oz	Little or none	0 mg	1	1	1	1	1
80 Proof Liquor	1 jigger	Little or none	0 mg	1	1	1	0	1
Chocolate Syrup	2 tbsp	Very high	38 mg	3	2	1	1	3
Fudge Sauce	2 tbsp	Very high	28 mg	3	2	1	0	3
Miso	1 cup	Very high	40 mg	3	2	1	3	2
Peanut Butter	1 tbsp	Very high	13 mg	3	3	1	3	1
Peanut Butter, reduced fat	1 tbsp	Very high	16 mg	3	3	1	2	1
Stuffing	1 cup	Very high	36 mg	3	2	2	2	3
Tahini	1 tbsp	Very high	16 mg	3	2	2	2	2
Cream Sauce, homemade	1 cup	Low	3 mg	1	1	2	1	1
Gravy	1 cup	Low	4 mg	1	2	0	1	1
Olive Oil & Vinegar		Little or none	2 mg	1	1	1	1	1
Soy Sauce	1 tbsp	Little or none	3 mg	1	2	1	0	3
Apple Butter	1 tbsp	Little or none	0 mg	1	1	1	1	1
Butter	1 pat	Little or none	0 mg	1	1	1	1	1
Catsup/Ketchup	1 packet	Little or none	1 mg	1	2	1	1	1
Cream Cheese	1 oz	Little or none	0 mg	1	1	1	1	1
Cream Cheese, low fat	1 oz	Little or none	1 mg	1	1	1	1	1
Cream Cheese, fat free	1 oz	Little or none	1 mg	1	1	1	1	1
Horseradish	1 tbsp	Little or none	0 mg	1	2	1	1	1
Jam/Jelly	1 tbsp	Little or none	1 mg	1	1	1	1	1
Italian Salad Dressing	1 tbsp	Little or none	0 mg	1	1	1	0	1
Mayonnaise	1 tbsp	Little or none	0 mg	1	1	1	1	1
Mustard, yellow	1 tsp or packet	Little or none	1 mg	1	1	1	1	1
Pancake Syrup	3/4 tbsp	Little or none	0 mg	1	1	1	0	1

Salsa	1 tbsp	Little or none	1 mg	1	1	1	1	1
Whipped Cream	2 tbsp	Little or none	0 mg	1	1	1	1	1
Whipped Topping	2 tbsp	Little or none	0 mg	1	2	1	1	1
All-Purpose Flour	1 cup	Very high	17 mg	3	2	1	2	2
Brown Rice Flour	1 cup	Very high	65 mg	3	2	1	1	3
Cocoa Powder	4 tsp	Very high	67 mg	3	3	1	3	3
Cornmeal	1 cup	Very high	64 mg	3	1	1	2	3
Soy Flour		Very high	94 mg	3	3	0	0	3
Soy Protein Isolate	1 oz	Very high	27 mg	3	2	1	3	1
White Rice Flour	1 cup	High	11 mg	3	1	1	2	1
Chili Powder	1 tbsp	Moderate	7 mg	2	2	1	0	1
Brewer's Yeast	1 tbsp	Moderate	7 mg	2	2	2	2	2
Corn Flour	1 cup	Low	3 mg	1	1	1	3	1
Cornstarch	1 cup	Low	3 mg	1	1	1	0	1
Lemon Juice, canned or bo	1 cup	Low	4 mg	1	1	1	0	2
Artificial Sweetener	1 packet	Very low	1 mg	1	1	1	0	1
Stevia Sweetener	1 tsp	Very low	42 mg	1	1	1	1	2
Bouillon Cube	1 cube	Very low	1 mg	1	1	1	1	1
Black Pepper	1 dash	Very low	0 mg	1	2	1	3	1
Barley Flour	1/2 cup	Very high	41 mg	3	2	1	3	3
Brown Sugar	1 cup packed	Very low	1 mg	1	1	1	1	1
Butter	1 pat	Very low	0 mg	1	1	1	0	1
Buttermilk	1 cup	Very low	1 mg	1	1	1	1	1
Corn Syrup, high fructose	1 tbsp	Very low	0 mg	1	1	1	0	1
Corn Syrup, light	1 tbsp	Very low	0 mg	1	1	1	0	1
Cod Liver Oil	1 tsp	Very low	0 mg	1	1	1	0	1
Cream Substitute	1 tsp	Very low	0 mg	1	1	1	0	1
Cream	1 tbsp	Very low	0 mg	1	1	1	1	1
Eggs	1 medium egg	Very low	0 mg	1	1	1	1	1
Eggbeaters	4 oz	Very low	0 mg	1	1	1	0	1
Garlic Powder	1 tsp	Very low	0 mg	1	2	1	1	1
Gelatin	1 tbsp or 1 envelo	Very low	0 mg	1	1	1	0	1
Honey	1 tbsp	Very low	0 mg	1	1	1	1	1
Lard	1 tsp	Very low	0 mg	1	1	1	0	1
Lemon Juice Raw, concent	1 tbsp	Very low	0 mg	1	1	1	0	1
Molasses	1 tbsp	Very low	0 mg	1	2	1	0	1
Oat Flour	1 cup	Very low	0 mg	1	2	1	3	1
Salt	1 tsp	Very low	0 mg	1	1	1	0	1
Shortening	1 tsp	Very low	0 mg	1	1	1	0	1
Sugar	1 tsp	Very low	0 mg	1	1	1	1	1
Sweet Whey, fluid	1 cup	Very low	1 mg	1	1	1	1	1
Sweet Whey, dried	1 tbsp	Very low	0 mg	1	1	1	0	2
Burritos with Beans	1 burrito	Very high	17 mg	3	2	0	3	3
Burritos with Beans & Meat	1 burrito	Very high	16 mg	3	2	0	3	3
Cheeseburger with Bun	1 burger & bun	Very high	13 mg	3	1	0	0	2
Chili with Beans	1 cup	Very high	24 mg	3	2	0	3	3
Enchilada with Cheese & B	1 enchilada	Very high	13 mg	3	1	0	0	3
Enchilada with Chicken	1 enchilada	Very high	13 mg	3	1	2	3	2
French Fries	4 oz	Very high	51 mg	3	2	1	3	2
Lasagna with Meat	1 serving	Very high	23 mg	3	2	2	0	3
Nachos with Cheese	6–8 chips	Very high	13 mg	3	1	1	0	3
Pizza with Cheese	2 slices	Very high	13 mg	3	2	2	0	2
Grilled Cheese Sandwich	1 sandwich	High	12 mg	3	1	1	0	2
Tacos	1 small taco	High	12 mg	3	1	1	0	2
Doughnut	1 doughnut	Moderate	5 mg	2	1	1	0	1
Eggroll	1 eggroll	Moderate	5 mg	2	2	1	1	2
Hot Dog with Bun	1 dog with bun	Moderate	9 mg	2	1	1	0	2
Onion Rings	6–8 rings	Moderate	5 mg	2	2	2	0	2
Chicken Nuggets	6 nuggets	Low	3 mg	1	1	1	1	1
Macaroni & Cheese	1 cup	Low	4 mg	1	2	2	0	1
Chicken Roll	1 package	Very low	1 mg	1	1	1	0	1
Clam Chowder	1 cup	Very high	13 mg	3	1	2	0	3
Lentil Soup	1 cup	Very high	39 mg	3	3	2	2	3
Miso Soup	1 cup	Very high	111 mg	3	1	1	3	3
Vegetable Beef Soup	1 cup	Moderate	5 mg	2	2	2	1	2
Chicken Noodle Soup	1 can	Low	3 mg	1	1	2	1	1
Cream of Wheat	1 cup	Very high	18 mg	3	1	1	1	3
Red River Cereal	1/4 cup	Very high	13 mg	3	2	2	2	3
Corn Grits	1 cup	Very high	97 mg	3	1	1	2	3

Farina Cereal	1 cup	Very high	16 mg	3	1	1	1	3
French Toast	2 slices	Very high	13 mg	3	2	1	2	3
Pancakes, homemade	4 pancakes	Very high	22 mg	3	1	1	2	3
Pancakes, dry mix	4 pancakes	Very high	37 mg	3	1	1	2	3
Danish Pastry, homemade	1 pastry	Very high	14 mg	3	1	2	2	3
Sweet Rolls, low fat	1 pastry	Very high	13 mg	3	1	2	2	1
English Muffins, whole whe	1 muffin	High	12 mg	3	2	2	2	2
Bran Muffins, store	1 muffin	Moderate	5 mg	2	2	2	2	2
Bran Muffin, low fat	1 muffin	Moderate	5 mg	2	2	2	2	2
Blueberry Muffins	1 muffin	Moderate	9 mg	2	2	2	1	2
Doughnut	1 doughnut	Moderate	5 mg	2	1	1	3	2
English Muffins, regular	1 muffin	Moderate	8 mg	2	1	2	2	2
English Muffins, multi-grain	1 muffin	Moderate	8 mg	2	2	2	2	2
English Muffins, wheat	1 muffin	Moderate	7 mg	2	2	2	2	2
Muffins, low fat	1 muffin	Moderate	5 mg	2	2	2	0	1
Pop Tart	1 tart	Moderate	7 mg	2	1	2	0	2
Cornbread	1 piece	Low	4 mg	1	1	1	1	1
Danish Pastry, fruit filled	1 pastry	Low	4 mg	1	2	2	0	1
Granola Bars, low fat	1 oz uncoated	Low	2 mg	1	2	2	1	1
Kashi Go Lean Bar	1 bar	Low	3 mg	1	2	1	2	1
Bacon	2 slices	Very low	0 mg	1	1	1	0	1
Carnation Instant Breakfast	1 packet	Very low	1 mg	1	1	1	0	1
Eggs	1 medium egg	Very low	0 mg	1	1	1	1	1
Eggbeaters	4 oz	Very low	0 mg	1	1	1	1	1
Granola Bars, hard & plain	1 bar	Very low	1 mg	1	1	2	0	1
Oatmeal Cereal	1 cup	Very low	0 mg	1	2	2	1	1
Pancake Syrup	3/4 tbsp	Very low	0 mg	1	1	1	0	1
All-Bran Original	1/2 cup	Very high	26 mg	3	3	2	3	3
All-Bran Buds	1/2 cup	Very high	20 mg	3	3	2	3	3
Complete Wheat Bran	3/4 cup	Very high	34 mg	3	3	3	3	3
Cracklin' Oat Bran	3/4 cup	Very high	15 mg	3	2	2	3	3
Frosted Mini-Wheats	1 cup	Very high	28 mg	3	2	2	3	3
Just Right Fruit & Nut	1 cup	Very high	13 mg	3	2	2	0	3
Granola with Raisins, low fa	2/3 cup	Very high	16 mg	3	2	2	1	3
Kashi Go Lean	3/4 cup	Very high	14 mg	3	2	2	3	3
Muesli Apple & Almond Cru	2/3 cup	Very high	20 mg	3	2	2	3	3
Muesli	2/3 cup	Very high	17 mg	3	2	2	3	3
Puffed Kashi	1 cup	Very high	13 mg	3	1	1	3	3
Raisin Bran	1 cup	Very high	46 mg	3	2	2	3	3
Raisin Bran Crunch	1 cup	Very high	27 mg	3	2	2	2	3
Raisin Squares Mini-Wheat	3/4 cup	Very high	41 mg	3	2	2	3	3
Smart Start	1 cup	Very high	15 mg	3	2	2	2	3
All-Bran with Extra Fiber	1/2 cup	High	11 mg	3	3	2	3	3
Cocoa Krispies	3/4 cup	High	11 mg	3	1	2	1	1
Kashi Good Friends	3/4 cup	High	10 mg	3	2	2	3	3
Complete Oat Bran Flakes	3/4 cup	Moderate	5 mg	2	2	3	2	1
Kashi Heart To Heart	3/4 cup	Moderate	8 mg	2	2	2	2	2
Healthy Choice Multi-Grain	3/4 cup	Moderate	7 mg	2	2	2	2	1
Froot Loops	1 cup	Low	2 mg	1	1	2	2	1
Honey Crunch Corn Flakes	3/4 cup	Low	3 mg	1	1	2	1	1
Rice Krispies	11/4 cup	Low	4 mg	1	1	1	1	3
Special K	3/4 cup	Low	3 mg	1	1	1	1	2
Special K, Red Berries	1 cup	Very low	2 mg	1	2	2	1	1
Special K, low carb	1/2 cup	Very high	35 mg	3	2	1	1	1
Smacks	3/4 cup	Low	3mg	1	1	2	1	1
Corn Flakes	1 cup	Very low	1 mg	1	1	1	1	1
Corn Pops	1 cup	Very low	1 mg	1	1	2	1	1
Crispix	1 cup	Very low	1 mg	1	1	2	1	1
Frosted Flakes	3/4 cup	Very low	1 mg	1	1	2	1	1
Product 19	1 cup	Very low	1 mg	1	1	2	1	1
100% Bran	1/3 cup	Very high	25 mg	3	3	3	3	3
40% Bran	3/4 cup	Very high	36 mg	3	2	1	3	3
Banana Nut Crunch	1 cup	Very high	25 mg	3	2	1	2	3
Cranberry Almond Crunch (	1 cup	Very high	35 mg	3	2	1	3	3
Fruit & Fiber Dates, Raisins	1 cup	Very high	41 mg	3	2	2	3	3
Great Grains Raisin, Dates	2/3 cup	Very high	17 mg	3	2	2	2	3
Great Grains Crunch Pecar	2/3 cup	Very high	18 mg	3	2	2	2	3
Grape Nuts	1/2 cup	Very high	14 mg	3	3	1	1	3
Original Shredded Wheat &	1 1/4 cup	Very high	53 mg	3	2	1	3	3

Blueberry Morning	1/2 cup	Moderate	8 mg	2	2	2	1	1
Grape Nuts Flakes	3/4 cup	Moderate	7 mg	2	2	2	1	2
Fruity Pebbles	3/4 cup	Low	2 mg	1	1	1	1	1
Honey Bunches of Oats wit	3/4 cup	Low	2 mg	1	2	1	1	1
Honey Bunches of Oats, Ho	3/4 cup	Low	3 mg	1	1	2	1	1
Honeycomb	1 1/3 cup	Very low	1 mg	1	1	1	1	3
Waffle Crisp	1 cup	Very low	1 mg	1	1	1	1	3
Basic 4	1 cup	Very high	17 mg	3	2	1	1	3
Fiber One	1/2 cup	Very high	13 mg	3	3	1	3	3
Honey Nut Clusters	1 cup	Very high	23 mg	3	2	3	1	3
Multi-Bran Chex	1 cup	Very high	36 mg	3	2	2	1	3
Nature Valley Cinnamon &	3/4 cup	Very high	13 mg	3	2	2	3	3
Oatmeal Crisp with Almond	1 cup	Very high	24 mg	3	2	2	1	3
Oatmeal Raisin Crisp	1 cup	Very high	13 mg	3	2	2	1	2
Raisin Nut Bran	1 cup	Very high	24 mg	3	2	2	1	3
Total Raisin Bran	1 cup	Very high	31 mg	3	2	2	1	3
Harmony	1 1/4 cup	High	11 mg	3	2	0	1	1
Wheaties Raisin Bran	1 cup	High	11 mg	3	2	2	1	3
Apple Cinnamon Cheerios	3/4 cup	Moderate	5 mg	2	1	1	2	2
Berry Burst Cheerios	1 cup	Moderate	7 mg	2	2	2	2	2
Cheerios	1 cup	Moderate	8 mg	2	1	1	2	2
Cinnamon Toast Crunch	3/4 cup	Moderate	5 mg	2	1	1	2	2
Corn Chex	1 cup	Moderate	5 mg	2	1	1	1	2
Count Chocula	1 cup	Moderate	5 mg	2	1	1	2	2
Frosted Cheerios	1 cup	Moderate	6 mg	2	1	2	2	2
Honey Nut Cheerios	1 cup	Moderate	7 mg	2	1	2	2	2
Golden Grahams	3/4 cup	Moderate	9 mg	2	1	1	2	2
Lucky Charms	1 cup	Moderate	5 mg	2	1	1	2	2
Reese's Puffs	3/4 cup	Moderate	8 mg	2	2	1	2	2
Team Cheerios	1 cup	Moderate	6 mg	2	2	1	2	1
Total Corn Flakes	1 1/3 cup	Moderate	5 mg	2	2	1	2	1
Wheat Chex	1 cup	Moderate	7 mg	2	2	1	1	2
Wheaties	1 cup	Moderate	8 mg	2	1	1	3	2
Whole Grain Total	1 1/4 cup	Moderate	8 mg	2	1	2	2	2
Cocoa Puffs	1 cup	Low	0 mg	1	1	1	3	1
Kix	1 1/3 cup	Low	1 mg	1	1	1	2	1
Rice Chex	1 1/4 cup	Low	0 mg	1	1	1	3	1
Trix	1 cup	Very low	0 mg	1	1	1	3	1
Low Fat 100% Natural Gran	3/4 cup	Very high	15 mg	3	2	2	1	3
100% Natural Granola Oats	1/2 cup	Very high	13 mg	3	2	2	1	3
Oat Bran	1 1/4 cup	High	10 mg	3	2	2	1	2
Honey Nut Oats	1 oz	Moderate	7 mg	2	2	0	1	2
Oatmeal Squares	1 cup	Moderate	5 mg	2	2	2	1	2
Puffed Wheat	1 1/4 cup	Moderate	9 mg	2	1	1	2	2
Toasted Oatmeal	1 oz	Moderate	6 mg	2	2	0	1	2
Puffed Rice	1 cup	Low	2 mg	1	1	1	1	1
Quaker Oat Cinnamon Life	3/4 cup	Low	3 mg	1	2	2	1	2
Quaker Oat Life	3/4 cup	Low	3 mg	1	2	2	1	2
Cap'n Crunch	3/4 cup	Very low	0 mg	1	1	1	1	1
Bran Flakes with Raisins, s	1 cup	Very high	57 mg	3	2	2	3	3
Nabisco Shredded Wheat	2 biscuits	Very high	42 mg	3	1	1	2	3
Nabisco Honey Nut Shredd	1 cup	Very high	47 mg	3	1	2	3	3
Spoon Size Shredded Whe	1 cup	Very high	45 mg	3	1	2	3	3
Uncle Sam	1 cup	High	11 mg	3	2	1	3	3
Just Right with Crunchy Nu	1 cup	Moderate	5 mg	2	2	2	2	2
Wheetabix Whole Wheat	2 biscuits	Moderate	8 mg	2	1	1	2	2
Healthy Valley Oat Bran Fla	1 cup	Very low	0 mg	1	2	2	1	3
